# Targeted Modulation of Interferon Response-Related Genes with IFN-Alpha/Lambda Inhibition

**DOI:** 10.3390/ijms23137248

**Published:** 2022-06-29

**Authors:** Debpali Sur, Katerina Leonova, Bar Levi, Shany Ivon Markowitz, Raichel Cohen-Harazi, Ilya Gitlin, Katerina Gurova, Andrei Gudkov, Albert Pinhasov, Igor Koman, Elimelech Nesher

**Affiliations:** 1 Department of Molecular Biology, Faculty of Natural Sciences, Ariel University, Ariel 4070000, Israel; debpali@ariel.ac.il (D.S.); barsh@ariel.ac.il (B.L.); albertpi@ariel.ac.il (A.P.); 2 Department of Cell Stress Biology, Roswell Park Comprehensive Cancer Center, Buffalo, NY 14263, USA; yekaterina.leonova@roswellpark.org (K.L.); ilya.gitlin@roswellpark.org (I.G.); katerina.gurova@roswellpark.org (K.G.); andrei.gudkov@roswellpark.org (A.G.); 3 Institute for Personalized and Translational Medicine, Ariel University, Ariel 4070000, Israel; shanyelbaz89@gmail.com (S.I.M.); raichelha@ariel.ac.il (R.C.-H.); igorko@ariel.ac.il (I.K.)

**Keywords:** IFN-α, IFN-λ, peptide, ALOS4, inflammation, cytokines

## Abstract

Interferon (IFN) signaling resulting from external or internal inflammatory processes initiates the rapid release of cytokines and chemokines to target viral or bacterial invasion, as well as cancer and other diseases. Prolonged exposure to IFNs, or the overexpression of other cytokines, leads to immune exhaustion, enhancing inflammation and leading to the persistence of infection and promotion of disease. Hence, to control and stabilize an excessive immune response, approaches for the management of inflammation are required. The potential use of peptides as anti-inflammatory agents has been previously demonstrated. Our team discovered, and previously published, a 9-amino-acid cyclic peptide named ALOS4 which exhibits anti-cancer properties in vivo and in vitro. We suggested that the anti-cancer effect of ALOS4 arises from interaction with the immune system, possibly through the modulation of inflammatory processes. Here, we show that treatment with ALOS4 decreases basal cytokine levels in mice with chronic inflammation and prolongs the lifespan of mice with acute systemic inflammation induced by irradiation. We also show that pretreatment with ALOS4 reduces the expression of IFN alpha, IFN lambda, and selected interferon-response genes triggered by polyinosinic-polycytidylic acid (Poly I:C), a synthetic analog of viral double-stranded RNA, while upregulating the expression of other genes with antiviral activity. Hence, we conclude that ALOS4 does not prevent IFN signaling, but rather supports the antiviral response by upregulating the expression of interferon-response genes in an interferon-independent manner.

## 1. Introduction

Inflammation is a complex process of sequential changes reflecting the response of cells and vascularized tissues to damage and is instigated by a variety of factors including viruses, bacteria, and chemicals [1,2]. The biological role of this process includes protection from the damaging agent as well as the initiation of the healing [3,4]. During inflammation, cytokines such as interferons (IFNs), chemokines, tumor necrosis factors (TNFs), and interleukins (ILs) are elevated in response to pathogen invasion and play a pivotal role in immune modulation [5,6]. On the other hand, the overexpression of cytokines leads to cellular stress and damage, known as a cytokine storm, characterized by the progression of the severity of the illnesses widely observed during viral entry [7,8]. For example, during the recent SARS-CoV-2 pandemic, enhanced disease severity and mortality [9,10] were observed following an increase in inflammatory cytokine-secreting cells including CD14 + CD16+ inflammatory monocytes in peripheral blood, and a dramatic increase in the levels of interleukins (IL1-β, IL2, IL7, IL8, IL9, IL10) and other cytokines (FGF2, GMCSF, IFNγ, MIP1α, MIP1β, PDGFB, VEGFA, G-CSF, IP10, MCP1 and TNFα) in the plasma of patients [11,12]. This phenomenon is a result of unrestrained immune response, which, along with increased inflammatory cell infiltration, leads to the excessive production of proteases and reactive oxygen species [13]. Damage caused by the cytokine storm leads to pathologies beyond the direct damage caused by the virus, resulting in decreased pathogen eradication and reduced chances for successful recovery [9,14]. Therefore, maintaining a balance in cytokine levels is necessary to preserve the organism’s immune homeostasis.

Inflammation in response to viral infection begins with the recognition of a virus by toll-like receptors (TLRs), retinoic-acid-inducible gene-I (RIG-I)-like receptors (RLRs), nucleotide oligomerization domain (NOD)-like receptors (NLRs), mitochondrial antiviral-signaling protein (MAVS), and cyclic GMP-AMP synthase (cGAS)–stimulator of interferon genes (STING), all components of the innate immune response [15]. The TLRs’ downstream signaling leads to the production of interferon (IFN) type I and type III through the activation of interferon regulatory 3/7 (IRF 3/7) transcriptional factors [16]. Both types of IFN act through STAT1 and STAT2 signaling, which initiate the expression of interferon-stimulated genes (ISG) controlled by the interferon-sensitive response element (ISRE) located in their promoters [17,18], followed by massive pro-inflammatory cytokine production, leading to an increase in vascular permeability and other physiological characteristics of the inflammatory response [19]. While type I and type III IFN-mediated signaling play an important role in controlling viral replication and critical regulatory functions affecting both the innate and adaptive immune responses [5,20], their dysregulation is associated with immune pathologies [21,22], including interferonopathies [23], for which there is a critical need for further research to understand IFN signaling and to discover effective means of modulation.

In recent years, independent research groups have demonstrated the potential use of peptides as anti-inflammatory agents due to their ability to both compete with pro-inflammatory molecules for specific binding sites [24,25,26], and penetrate the cell and trigger or inhibit signal transmission [27,28]. Using phage display [29], we discovered a 9-amino-acid cyclic peptide targeting α_v_β_3_ integrin-mediated signaling and named it ALOS4. Studying the role of ALOS4 in malignancy, we found that this peptide exhibits anti-cancer properties in vivo and in vitro [29,30]. Specifically, the ALOS4 treatment of animals with subcutaneous murine and human melanoma leads to the inhibition of tumor growth over a wide therapeutic window with no toxicity [29,31]. Based on our recent results [31], we suggested that the anti-cancer effect of ALOS4 may be achieved through its anti-inflammatory properties. To confirm our hypothesis, we explored the effect of the ALOS4 peptide on both acute and chronic inflammation using in vitro and in vivo models. In this paper, we present a comprehensive analysis of the anti-inflammatory properties of ALOS4 mediated by the selective modulation of interferon-stimulated genes.

## 2. Results and Discussion

**Figure 1:**
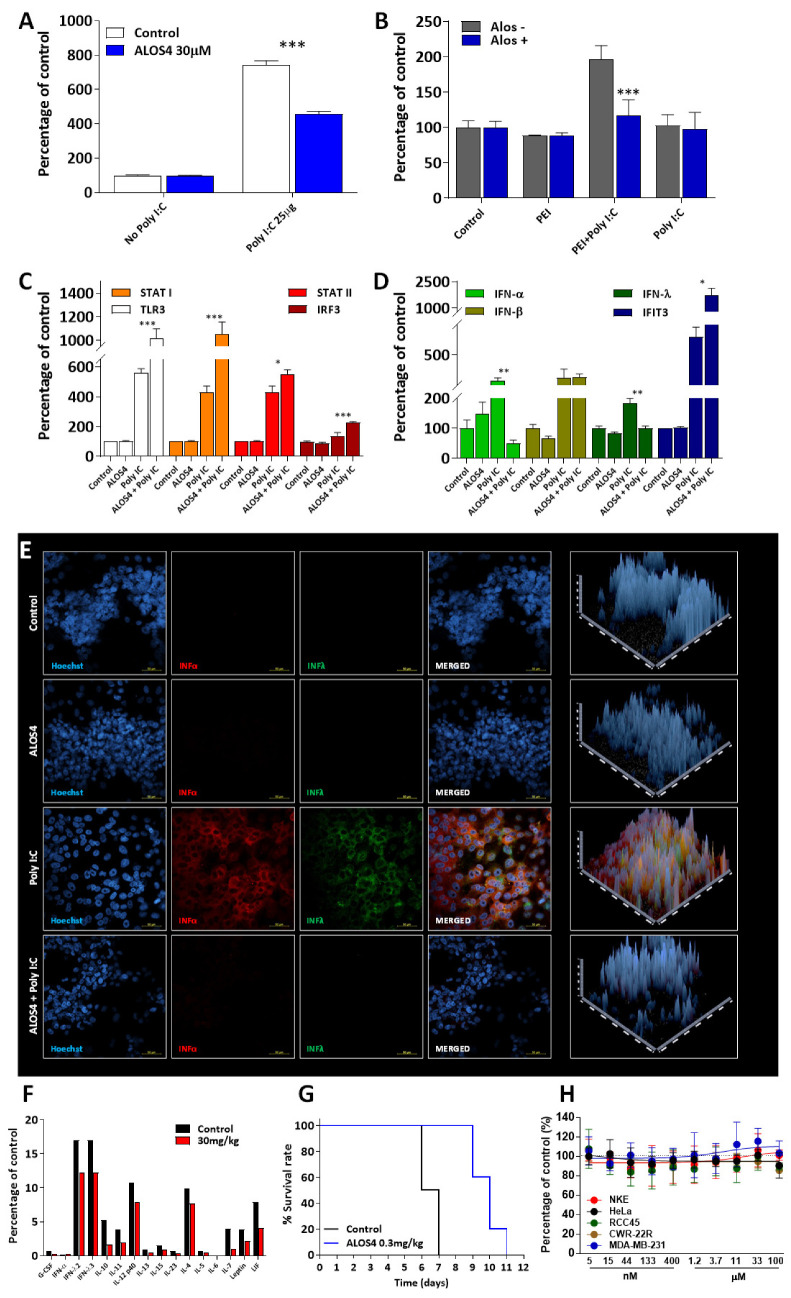
ALOS4 suppresses pro-inflammatory signaling. (**A**) A synthetic analog of viral dsRNA (Poly I:C, 25 µg/mL) was used to induce interferon type I signaling in HT1080-ISRE-mCherry cells. IFN response was suppressed with 30 µM ALOS4 pretreatment for 48 h. Multiple reads (8 per well) at 574_Ex_/610_Em_. (**B**) Pretreatment with 0.3 µM of ALOS4 HCT116-ISRE-Luc cells in the presence of 0.1 µg/mL PEI transfection reagent and Poly I:C, 25 µg/mL was analyzed by luminescence measurement using Bright-Glo™ Luciferase Assay System. (**C**–**F**) Changes in the expression of antiviral and interferon-sensitive genes (ISG) (**C**,**D**), and type I and III IFNs themselves (**D**) in HT1080 cells evaluated by qPCR (**C**,**D**), and by immunofluorescent staining and a 3D projection of IFN-α and IFN-λ as detected by confocal analysis before and after pretreatment with 0.3 µM ALOS4 for 48 h followed by 25 µg/mL of Poly I:C. (**C**,**D**) Changes in expression of antiviral and interferon-sensitive genes (ISG) in HT1080 cells (**C**,**D**), and type I and III IFNs themselves (**D**), evaluated by qPCR before and after pretreatment with 0.3 µM ALOS4 for 48 h followed by 25 µg/mL of Poly I:C; (**F**) ALOS4 decreases serum cytokine levels. Data are shown for mice with chronic stress and inflammation, treated sub-chronically with 30 mg/kg of ALOS4 compared to control mice treated with saline. Each column represents the pool of serum from 10 mice. (**G**) Daily intraperitoneal treatment with 0.3 mg/kg of ALOS4 increased survival of p53 KO mice irradiated with 12 Gy by more than 50%. (**H**) Analysis of ALOS4 cytotoxicity. The viability of cells treated with different doses of ALOS4 was determined after 72 h of incubation using a resazurin-based assay. Error bars represent the mean ± SD. Significance was assessed using one-way ANOVA with Tukey’s means separation test, indicated by (*) at *p* < 0.05, (**), at *p* < 0.01, and (***) at *p* < 0.001.

To assess the effect of ALOS4 on IFN activation, we employed an assay with two reporter constructs encoding either mCherry fluorescent protein or luciferase (Luc) enzyme under the control of an ISRE element [32] activated by polyinosinic-polycytidylic acid (Poly I:C), a synthetic analog of viral double-stranded RNA (dsRNA). We showed that the pretreatment of HT1080-ISRE-mCherry cells with ALOS4 for 48 h downregulates the type I IFN response (Figure 1A; one-way ANOVA: F [3,92] = 420.9, *p* < 0.0001, followed by Tukey’s means separation test (ALOS4 + Poly I:C vs. Poly I:C, *p* < 0.0001)). Similarly, pretreatment of HCT116-ISRE-Luc cells with ALOS4 causes the suppression of luciferase expression (one-way ANOVA: F [7,16] = 16.19, *p* < 0.0001, followed by Tukey’s means separation test (PEI + ALOS4 + Poly I:C vs. PEI + Poly I:C, *p* = 0.0002)) induced by Poly I:C (Figure 1B). Together, these results support the hypothesis that ALOS4 downregulates pro-inflammatory interferon synthesis.

Furthermore, we demonstrated in vitro that in the presence of ALOS4, Poly I:C significantly upregulates the expression of genes with specific antiviral activity, including: TLR3, STAT1, STAT2, IRF3, and IFIT3 (Figure 1C,D; one-way ANOVA, followed by Tukey’s means separation test for ALOS4 + Poly I:C vs. Poly I:C: TLR3, *p* = 0.0003; STAT1, *p* = 0.0002; STAT2, *p* = 0.037; IRF3, *p* = 0.0004, and IFIT3 *p* = 0.0308). Elevated TLR3 expression indicates the recognition of the dsRNA mimetic by the cell, and the subsequent activation of TLR3 downstream signaling indicates that ALOS4 does not prevent the entry of the virus-like agent. At the same time, pretreatment with ALOS4 affects the expression of IFN-α and IFN-λ (Figure 1D; one-way ANOVA, followed by Tukey’s means separation test for ALOS4 + Poly I:C vs. Poly I:C: IFN-α, *p* = 0.0058; IFN-λ, *p* = 0.0011) and stimulates a strong increase in interferon-induced protein with tetratricopeptide repeats 3 (IFIT3), known for its important role in antiviral signaling [33], possibly via IRF3 activation (Figure 1C). This hypothesis is supported by the previously described phenomenon that, independent of interferon, IRF3 induces the upregulation of interferon-stimulated genes in a human CMV model [34]. The observed differences in the expression patterns of IFN-α and IFN-β in the presence of ALOS4 may be explained by the differential binding affinity of these interferons to their shared receptors [35]. The ALOS4-triggered downregulation of the IFN-α and IFN-λ mRNA expression induced by Poly I:C treatment was also accompanied by a decrease in the protein level of these interferons, visualized by immunofluorescent staining (Figure 1E).

In a murine model of chronic inflammation [36], type I IFN levels are generally unaffected while IFN-λ, as well as interleukins and other cytokines, are elevated in blood. We showed that despite the fact that sub-chronic treatment (IV injections, every second day for 10 days) with 30 mg/kg of ALOS4 did not affect type I IFN expression, the levels of IFN-λ, interleukins, and other cytokines were reduced as compared to the control group (Figure 1F). Since ALOS4 was discovered through specific binding to integrin α_v_β_3_ [29,30], we may speculate that the anti-inflammatory properties of ALOS4 are also associated with the α_v_β_3_ signaling effect on cytokine synthesis in general [37] and type I IFN in particular [38].

The p53 gene is a known suppressor of the inflammatory response, DNA damage regulator, and modulator of human TLRs gene expression [38,39]. We used p53 KO mice to assess the effect of ALOS4 treatment on inflammation caused by acute radiation [40]. We found that the survival time of p53 KO mice treated with ALOS4 was almost two-fold longer as compared to the untreated group (Figure 1G; Gehan–Breslow–Wilcoxon test, *p* = 0.0004). Since p53 is known to be involved in the regulation of epigenetic silencing of endogenous retroelements, p53 KO mice may be characterized by a lack of epigenetic integrity and the development of inflammation through the IFN response [32]. Moreover, due to the demonstrated pro-cancer activity of type I IFN following radiation via CD8+ T cell-mediated cytotoxicity protection [41], as well as recent evidence demonstrating that prolonged exposure to type I IFN leads to immune exhaustion allowing cancer cells to escape immune cell surveillance [42,43], we may assume that the previously demonstrated anti-cancer effect of ALOS4 [29,31] in the absence of any cytotoxicity (Figure 1H) is elicited by the inhibition of IFN types I and III. Hence, we propose further investigation of the hypothesis that ALOS4 indirectly influences epigenetic mechanisms and thus modulates IFN types I and III signaling in response to the inflammatory response.

## 3. Materials and Methods

### 3.1. Compounds

#### 3.1.1. ALOS4

ALOS4 cyclic peptide (H-cycl (Cys-Ser-Ser-Ala-Gly-Ser-Leu-Phe-Cys)-OH) was synthesized by Shanghai Hanhong Scientific (Cat# P120301-LG221431; Shanghai Hanhong Scientific, Shanghai, China). For in vitro experiments, ALOS4 was dissolved in saline and stored at −20 °C at 10 mM stock concentration. Prior to experimentation, ALOS4 was thawed and further diluted to working concentrations of 0.3, 0.5, 1 and 30 µM. For in vivo experiments, ALOS4 was dissolved in saline and stored at −20 °C at 300 mg/kg concentration. Prior to experimentation, ALOS4 was thawed and further diluted to working concentrations, of 0.3 or 30 mg/kg.

#### 3.1.2. Polyinosinic-Polycytidylic Acid (Poly I:C)

Poly I:C (Cat# tlrl-pic; InvivoGen, San Diego, CA, USA) was prepared in saline by resuspending and heating to 50 °C at a concentration of 2 mg/mL to ensure complete solubilization followed by natural cooling to room temperature for proper annealing of double-stranded RNA and stored at −20 °C until use.

### 3.2. Cells

HT1080 (human fibrosarcoma, Cat# CCL-121), HCT116 (human colorectal carcinoma, Cat# CCL-247), HeLa (human cervical carcinoma, Cat# CCL-2), CWR-22R (human prostate carcinoma, Cat# CRL-2505), and MDA-MB-231 (human mammary gland ade-nocarcinoma, Cat# CCL-247) were purchased from ATCC (Manassas, VA, USA). RCC45 (human renal cell carcinoma) and NKE (human normal kidney epithelium) were pro-vided by Katerina Gurova (Roswell Park Comprehensive Cancer Center, Buffalo, NY, USA). In addition, HT1080 cells transfected with mCherry reporter under an ISRE (interferon-sensitive response element) promoter and HCT116 with Luc reporter under ISRE promoter were used. The cells were cultured in an appropriate media: Dulbecco’s Modified Eagle Medium (DMEM) (Cat# 41965-039; Fisher Scientific (Gibco), Grand Island, NY, USA) or Roswell Park Memorial Institute (RPMI) 1640 Medium (RPMI) (Cat# 21875034; Fisher Scientific (Gibco), Grand Island, NY, USA) with 4.5 g/L glucose and L-glutamine augmented with 10% FBS (Cat# 16000-036; Gibco, Grand Island, NY, USA), 100 U/mL penicillin and 100 μg/mL streptomycin in a humidified atmosphere at 37 °C with 5% CO_2_.

### 3.3. Cytotoxicity

HeLa, MDA-MB-231, CWR-22R, RCC45, and NKE cells were seeded as 3 × 10^3^ cells/well in 96-well plates for overnight attachment. Cells were treated for 72 h with a range of drug concentrations. Cell viability was determined with resazurin saline solution. Fluorescence was measured at 560_Ex_/590_Em_. A total of 50 μM of 9-aminoacridine was used as a positive control for complete cell death.

### 3.4. Animals

Eight-week-old submissive mice (*n* = 20 total) representing a model of chronic inflammation [36], as well as their wild-type background strain Sabra mice [44], maintained in the Ariel University animal facility were used for cytokine analysis. All mice were maintained under a 12 h light/12 h dark cycle with Purina rodent chow (Cat# 2060, Envigo, Ness-Ziona, Israel) and water provided ad libitum. The mice were randomly divided into two groups (10 mice/group): sub-chronically treated by IV injections, every second day for 10 days, with 30 mg/kg of ALOS4 (*n* = 10) and saline (*n* = 10; negative control). Random numbers were generated using the standard = RAND() function in Microsoft Excel. Mice were housed five per cage in a room maintained at 22 ± 0.5 °C. For each cage, two different investigators were involved as follows: the first investigator (EN) prepared syringes with the same treatment/cage based on the randomization table. The second investigator (BL), responsible for the administration, was blind to the treatments the mice received. After the experiment, the mice were euthanized with CO_2_ and blood samples were collected for further cytokine analysis, as a pool of serum from 10 mice for each experimental group. Due to the pooled nature of further cytokine analysis, the experiment could not be blinded at the analytic stage. All procedures with animals were performed after review and approval by the Institutional Animal Care and Use Committee of Ariel University (IL-123-02-17; Ariel, Israel).

Seven-week-old p53 KO c57bl/6 mice were used to assess the effect of ALOS4 treatment on inflammation caused by acute radiation [45]. Mice (*n* = 9 total) were randomly divided into two groups: IP injected with ALOS4 (*n* = 5) or saline (negative control; *n* = 4) injections. Treatment was started 2 days prior to irradiation with 12 Gy and continued every day until the termination of experiment, after the death of animal. Survival statistics were obtained using Gehan–Breslow–Wilcoxon test (GraphPad Prizm 7 software). For each group, two different investigators were involved as follows: the first investigator (EN) prepared a syringe with same treatment/cage based on the randomization table. The second investigator (IG), responsible for the administration, was blind to the treatments the mice received. Random numbers were generated using the standard = RAND() function in Microsoft Excel. Animal care and treatment were in compliance with NIH protocols approved under the Institutional Animal Care and Use Committee of Roswell Park Cancer Institute (1081 M; Buffalo, NY, USA).

### 3.5. ISRE Response

HT1080-ISRE-mCherry cells were plated in 6-well plates at 2 × 10^4^ cells/well. The next day, DMEM was replaced with DMEM containing ALOS4 or saline as control and cells continued to grow for 48 h. Following the 48 h incubation, 25 µg/well of Poly I:C was added for an additional 16 h incubation. Following treatment, cells were subjected to fluorescence quantification as follows: cells were washed with 1× PBS, trypsinized and resuspended in 300 µL of 1× PBS, and 200 µL of cell suspension was further seeded into 96-well black-walled, clear-bottom plates. The intensity of the mCherry fluorescence signal was detected with multiple reads per well at 574_Ex_/610_Em_ using ClarioStar plate reader (BMG Labtech, Ortenberg, Germany).

HCT116 cells were plated in 96-well plates at 3 × 10^3^ cells/well. ALOS4, Poly I:C or saline as control were administered with/without PEI—transfection reagent. In order to detect reporter signal, the media were removed, and 30 µL of Bright-Glo™ Luciferase Assay System (Cat# E2610; Promega, Madison, WI, USA) was added. The signal was quantified by ClarioStar plate reader (BMG Labtech) on luminescence parameters.

### 3.6. Cytokines Analysis

Blood samples of treated mice were collected by terminal bleeding from the heart and centrifuged at 2000× *g* for 15 min. Serum was removed to a new tube and stored at −20 °C for future use. Mouse XL Cytokine Array Kit (Cat# ARYO28, R&D) was used to detect the cytokines in the serum of mice according to the manufacturer’s instructions. A pool of serum from 10 mice for each experimental group was prepared. Streptavidin–HRP and Chemi Reagent Mix were used to detect the antibodies bound to the membranes with samples. The membranes were then exposed (Image Quant LAS4000 mini; GE Healthcare, Milwaukee, WI, USA) and analyzed using ImageQuant TL software (GE Healthcare).

### 3.7. Gene Expression

Total RNA was extracted from HT1080 cells using the Zymo Research RNA miniprep kit (Cat# R1018, Irvine, CA, USA). DNase treatment was performed using on-column DNase digestion. RNA concentration was measured at 260 nm using NanoDrop spectrophotometer (Thermo Scientific, Wilmington, DE, USA, Cat# DE19810) and 260/280 ratio method was used to verify that the samples met proper purification standards, around 2. A total of 1 μg of total RNA was reverse-transcribed using a reverse-transcription system (Promega, Cat# A3500, Madison, WI, USA). The master mix for cDNA synthesis consisted of 10× reverse-transcription buffer, dNTP mix, oligo (dT) (18T) primers, and AMV enzyme. The reverse-transcription reaction was performed in a thermocycler (Bio-Rad Laboratories, T100, Hercules, CA, USA) using a two-step program: 42 °C for 60 min followed by heating to 70 °C for 15 min to terminate the reaction, and maintained at 4 °C. The quantitative RT-PCR was performed using 2× PCR SYBR Green Master Mix (Applied Biosystems, Cat# 4344463, Warrington, UK), with a 100 nM mixture of forward and reverse primers (TLR3 forward: agtgccgtctatttgccaca and reverse: gcatcccaaagggcaaaagg; STAT1 forward: atggtgcatcatgggcttca and reverse: catggaagtcaggttcgcct; STAT2 forward: ggacccccatcagaccaaag and reverse: atgcagggctgggtttctac; IRF3 forward: ccctctgagaacccactgaa and reverse: tgcctcacgtagctcatcac; IFIT3 forward: tgcagggaaacagccatcat and reverse: aggcgtagtttccccaagtg; IFN-α forward: aactcccctgatgaatgcgg and reverse: tagcaggggtgagagtctttg; IFN-β forward: aatgggctacaccgaagcaa and reverse: ttgcggaaggatgtctccac; IFN-λ forward: tggtgactttggtgctaggc and reverse: ggccttcttgaagctcgcta; as well as Actin-β used as an endogenous normalization factor, forward: gggcatgggtcagaaggatt and reverse: actccatgcccaggaaggaa), 4 µg of cDNA and RNase/DNase free water. Samples were placed in real-time PCR (AriaMx; Cat# G88230A, Santa Clara, CA, USA) and reactions were performed in a thermocycler: 180 s at 95 °C, followed by 40 cycles of 3 s at 95 °C and 30 s at 60 °C.

### 3.8. Immunofluorescent Staining

Cells were plated in 35 mm glass bottom plates from MatTek Corporation (Ashland, MA, USA). After treatment, cells were washed with 1× PBS and fixed in 4% paraformaldehyde at room temperature for 15 min. Cells were then washed three times with 1× PBS. Blocking was completed in 3% BSA, 0.1% Triton-X100 in 1× PBS. Anti-interferon alpha (Abcam, Cat# ab196221) and Anti-IL-28A (Abcam, Cat# ab233754) antibodies were used to detect IFN-α and IFN-λ proteins, respectively. AlexaFluor 488 donkey anti-mouse (Invitrogen, Cat# A21206) and AlexaFluor 594 donkey anti-sheep (Jackson ImmunoResearch, cat# 713-585-147) were used as secondary antibodies. Antibodies were diluted in 0.5% BSA + 0.05% Triton X100 in 1× PBS. After each antibody incubation, cells were washed three times with 0.05% Triton X100 in 1× PBS. For DNA counterstaining, a 1 µg/mL solution of Hoechst 33342 (Cat# H1399, Sigma-Aldrich, St. Louis, MO, USA) in 1× PBS was used. Immunofluorescence images were acquired with a 100× oil-immersion lens using the Zeiss confocal microscope (LSM700, Opti-Ups 1000B).

## 4. Conclusions

Healing and survival following a viral infection depend on the ability of the body to control the inflammatory response, including the prevention or attenuation of the cytokine storm and the consequences and complications driven by acute inflammation. The cyclic peptide ALOS4 selectively reduces the expression of interferon response genes, attenuates cytokine levels, and consequently leads to a reduction in inflammation. We believe that comprehensive analysis of the protective effect of ALOS4 on the modulation of inflammation will shed light on the detailed mechanism of its action mediated by immune system response regulation.

## 5. Patents

Pinhasov A. has an ALOS4 patent (62/127,854). Leonova K., Gudkov A., Pinhasov A., Koman I., and Nesher E., have a related patent (US Patent App. 17/068,849).

## Data Availability

All data generated or analyzed during this study are included in this published article. The raw data are available from the corresponding author on reasonable request.
